# Kruppel-Like Factor 4 Regulates Granule Cell Pax6 Expression and Cell Proliferation in Early Cerebellar Development

**DOI:** 10.1371/journal.pone.0134390

**Published:** 2015-07-30

**Authors:** Peter Zhang, Thomas Ha, Matt Larouche, Douglas Swanson, Dan Goldowitz

**Affiliations:** Centre for Molecular Medicine and Therapeutics, Child and Family Research Institute, Department of Medical Genetics, University of British Columbia, 950 West 28th Avenue, Vancouver, BC, V5Z 4H4, Canada; University of Kentucky, UNITED STATES

## Abstract

Kruppel-like factor 4 (Klf4) is a transcription factor that regulates many important cellular processes in stem cell biology, cancer, and development. We used histological and molecular methods to study the expression of Klf4 in embryonic development of the normal and Klf4 knockout cerebellum. We find that Klf4 is expressed strongly in early granule cell progenitor development but tails-off considerably by the end of embryonic development. Klf4 is also co-expressed with Pax6 in these cells. In the Klf4-null mouse, which is perinatal lethal, Klf4 positively regulates Pax6 expression and regulates the proliferation of neuronal progenitors in the rhombic lip, external granular layer and the neuroepithelium. This paper is the first to describe a role for Klf4 in the cerebellum and provides insight into this gene’s function in neuronal development.

## Introduction

The cerebellum, which represents about 10% volume of the brain, consists of more than half of all neurons in the brain. The numerous cerebellar neurons belong to only a few neuronal groups and are arranged in a simple and well-defined cytoarchitectural organization[1]. However, development of the seemingly “simple” cerebellum requires a precise spatial and temporal regulation of different cellular processes. The cerebellar neurons can be grouped by their neurotransmitters: the excitatory neurons utilizing glutamate and the inhibitory neurons utilizing GABA. The glutamatergic granule cells (GCs) and other excitatory neurons and the GABAergic Purkinje cells (PCs) and inhibitory interneurons are born from two distinct neurogenic regions at different developmental stages; through tightly regulated developmental processes, these neurons proliferate, differentiate, migrate and interact to produce a mature and functional cerebellum. Thus, the cerebellum serves as an excellent model for neurodevelopmental research because it is structurally simple, yet the cerebellar neurons undergo all the major developmental events, such as cell specification, differentiation, proliferation and migration, that are critical and common to development of the central nervous system, in general.

The glutamatergic GC precursors are generated from a germinal epithelium known as the rhombic lip at around embryonic day 10 (E10). The rhombic lip progenitors, located lateral and caudal to the NE, are specified by the bHLH transcription factor, Math1 (also known as Atoh1)[2,3]. The GC precursors, which give rise to differentiated GCs, most actively proliferate from E15 to post-natal day 8 (P8)[2,4]. At E12, the GCs begin their first migration to cover the dorsal surface of the cerebellum forming the external granular layer (EGL). It is not until the time of birth that the GCs begin their second migration into the cerebellar parenchyma to their final destination where they form the IGL. The EGL ceases to exist at around P21[1]. The paired-box transcription factor, Pax6, is strongly expressed in the GC throughout the course of development [5]. Pax6 is important for GC development[6]; however its regulatory pathway is not well understood; thus, we are interested in identifying genes that regulate, or are regulated by, Pax6 because these genes might play important roles during cerebellar development through interactions with Pax6.

Klf4 was bioinformatically identified as a transcriptional regulator for Pax6 using Enhancer Element Locater (EEL)[7]. Klf4 is one of the four genes necessary to create an induced pluripotent stem cell and has been extensively studied for its role in cell proliferation, differentiation and survival in multiple cell types[8] and its association with Pax6 has been documented in corneal development[9,10]. However, the role of Klf4 in cerebellar development remains unknown. We hypothesized that Klf4 is a key transcription factor for cerebellar development as a regulator of Pax6.

Klf4 belongs to the Kruppel-like factor family, which contains three C-terminal C2H2-type zinc fingers that bind DNA. The name “Kruppel-like” comes from its strong homology with the *Drosophila* gene product Kruppel, an important gene in segmentation of the developing embryo. Klf4 has been studied for its roles in stem cell maintenance, oncogenesis and embryonic development. Klf4 is one of the four genes necessary to create an induced pluripotent stem cell; although the mechanism of Klf4 in the self-renewal of the stem cell remains unclear, it is speculated that it might function to maintain cell proliferation[11] or inhibit apoptosis[12]. Klf4 also plays important roles in tumorigenesis—depending on tissue and environment, it can function as an oncogene as the over-expression of Klf4 could repress expression of p53 through the Ras/P21 pathway[13], which would prevent cell apoptosis. On the other hand, Klf4 can also function as a tumor suppressor as it can antagonize the Wnt pathway resulting in the inhibition of cell proliferation. Lastly, Klf4 is an important transcription factor for homeostasis of multiple tissue types. It is essential for the differentiation of goblet cells in the colon as knocking out Klf4 resulted in the absence of these cells [14]. Klf4 is also critical for development of the granular layer of the skin[15]; the Klf4-/- mouse dies several hours after birth due to the defective body barrier which causes extensive loss of body fluid[15]. The function of Klf4 in brain development has been studied through myc-activated overexpression where cell proliferation and differentiation are inhibited along with defects in cilia genesis that lead to hydrocephalus[16].

Klf4 has been identified as a cancer suppressor gene that is frequently inactivated in medulloblastoma [17]–a tumor that oftentimes originates from cerebellar granule neurons. However, the role of Klf4 in normal cerebellar development has not been studied. Here we report our findings of roles of Klf4 during cerebellum development. We find that Klf4 is important for granule cell proliferation through E13.5 and E15.5; we also find that Pax6 expression is lowered in the Klf4-/- cerebellum and we find that Klf4 acts as an upstream regulator of Pax6.

## Methods

### Expression analysis and transcription factor binding site prediction

Two databases were used to access quantitative expression of genes of interest in the cerebellum: 1) CbGRiTS is a time-course, microarray database constructed from transcriptomes of mouse cerebellum from E12 to P9 [18]. 2) RIKEN FANTOM5 contains 5’cap sequencing, time-course data of mouse cerebellar transcriptome from E12 to P9) [19]. To examine the spatial expression of genes of interest in the developing mouse cerebellum, histological data of various cerebellar transcription factors in wild-type mouse were obtained from the online Genepaint (www.genepaint.org) and Allen Brain Atlas (www.brain-map.org) databases.

To determine if there were any transcription binding sites in genes with strong granule-cell expression, Genbank (http://www.ncbi.nlm.nih.gov/genbank) sequence data from 200 Kbp upstream of the transcript start site were analyzed with Enhancer Element Locator (EEL) software [7]. The detailed methods for EEL have been described elsewhere [20]; the values for parameters Lambda, Xi, Nu, Mu and Nucleotides Per Rotation were set at the default setting for the program: 2.0, 200.0, 200.0, 0.5 and 10.4, respectively. Two statistical cut-offs were used for Enhancer Element Locator analysis: a p-value less than 0.001, which represents the significance that a binding site is over-represented upstream of Pax6 when compared with whole genome background; and a confidence level of 92%, which measures the number and conservation of binding sites found upstream of Pax6[7].

### Klf4 colony maintenance and breeding

Canadian Council on Animal Care approved this research of ethical approval (approval number- A12-0190). The research was conducted in accordance with these policies and all efforts were made to minimize suffering. Klf4-null mice (Klf4-/-) were a gift from Elaine Fuchs’ Lab at Rockefeller University; they were housed at University of Chicago accredited by Association for Assessment and Accreditation of Laboratory Animal Care (AAALAC) with approval by Animal Care and Use Committee (approval number—NHGRI ACUC 08–0059). The knock-out of Klf4 was achieved by a substitution of the entire exon 2 and 3 as well as part of exon 1 of the Klf4 gene with a neomycin sequence (neo) on a C57BL/6 background; this results in the elimination of expression of Klf4 transcripts and protein products[15]. We recovered heterozygous Klf4 animals with in-utero injection of frozen embryos. The colony was maintained in a heterozygous state that showed normal cerebellar and behavioral development. Klf4 knockout mice are perinatal lethal due to defect in skin development which causes excess loss of body fluid shortly after birth[15]. The Klf4-/- embryos were generated with time pregnancy and collected at embryonic stages (E13.5-E18.5).

### Histological methods and analysis

Transcardiac perfusion with PBS and 4% paraformaldehyde was used to prepare animal tissues after deep Avertin anesthesia. Tissues were post-fixed *in situ* for 2 hrs in 4% PFA. The brains were dissected out and stored at 4°C in PBS (0.1 M, containing 0.02% Na Azide) until processing. Tissues were cryoprotected in 30% sucrose in PBS overnight or until they sunk to the bottom of the solution and serially cryosectioned at 12–16 μm. Serial sections from brains were stained with cresyl violet for histology. Single-label immunofluorescence staining of the tissue was carried out as previously described: anti-Klf4 (AF3158, goat, R&D Systems, 1:200), to detect Klf4 expression in the wild-type animals; anti-Pax6 (PRB-278P, rabbit, Covance, 1:200), to highlight wild-type granule cells and granule cell precursors; anti-Calbindin D-28k (AB1778, rabbit, Chemicon, 1:200), to detect Purkinje cell soma and dendrites; anti-Pax2 (71–6000, rabbit, Invitrogen, 1:200), to identify interneuron precursors; and anti-Gfap(sc-51908, mouse, Santa Cruz, 1:200), anti-Glast(MABN794, goat, Millipore, 1:200), to identify cerebellar glial cells. Anti-Klf2(bs-2772R, rabbit, Bioss, 1:200), anti-Klf5(bs-2385R, rabbit, Bioss, 1:200) and anti-β catinen(ab6302, rabbit, abcam, 1:200) were used for further investigation of the Klf4 pathway. The secondary antibodies used for these analyses were donkey anti-goat (95382, Jackson ImmunoResearch, 1:200), goat anti-Rabbit whole IgG Alexa 594 conjugate (A11012, 1:200) and goat anti-mouse F(ab′b_2_ Alexa 594 conjugate (A11020, 1:200) (Molecular Probes/Invitrogen). Analysis and photomicroscopy of brightfield histochemistry was performed with a Zeiss Axiophot microscope with the Axiocam/Axiovision hardware-software components (Carl Zeiss).

Two quantitative analyses were conducted on histological sections: the number of Pax6+ cells in EGL and the number of BrdU+ in three regions of the cerebellum (the rhombic lip, the EGL, and the neuroepithelium). Cell counts were started at the first appearance of the external granular layer in the cerebellum, and at every 10th sagittal section that followed, throughout both sides of the cerebellum. The Klf4-null cerebellum was compared against the wild-type cerebellum using one-tailed Students’ T-test. p<0.05 was considered a significant difference in cell number between the two groups.

### Assays for cell proliferation and cell death

To examine cell proliferaton, mice were injected with 50 mg BrdU/kg 60 minute before to perfusion with a 3:1 70% EtOH:acetic acid. For anti-BrdU immunohistochemistry, brains were embedded in paraffin, sectioned on a microtome at 16um, and mounted on glass slides. Sections were deparaffinized, rehydrated, and treated with 1M HCl for 30 minutes at 37°C. Then, the slides were incubated with mouse anti-BrdU monoclonal antibody (1:200 dilution; BD Biosciences, Mississauga, ON, Canada) overnight followed by incubating in biotinylated horse anti-mouse immunoglobulin (1:200 dilution; Vector Laboratories, Burlingame, CA, USA) for 1 hour on the next day. Lastly, the slides were stained using the VECTASTAIN Elite ABC kit (Vector Laboratories) and 3, 30-diaminobenzidine (SigmaeAldrich). For assessing cell death, the mice were processed for perfusion and the brains for sectioning as described for the cell proliferation work, above. The slides were immuno-stained with ApopTag Plus Fluorescein In Situ Apoptosis Detection Kit (S7111, ApopTag FITC-direct, Chemicon).

### Real-time PCR

cDNA from Klf4-null and wild-type littermates were produced with random hexamers using the High Capacity cDNA Archive kit (Applied Biosystems). cDNA products were diluted to 100 ng total RNA input. Sequences of the transcript of interest were loaded into Primer Express software (Applied Biosystems). Amplicon lengths were between 75 and 125 bp. The qPCR was performed with the FAST SYBR Green PCR Master Mix (Applied Biosystems) on an ABI StepOne Plus Sequence Detection System (Applied Biosystems). All runs were normalised to 18s RNA. Three biological replicates were prepared for each gene target and three technical replicates were performed for each biological replicate. Gene expression was represented as relative quantity against the negative control which used water as the template (noted as “Relative Quantity vs. H2O” in Figs).

The results of Real-Time PCR were analyzed and graphed by ABI StepOne Plus Sequence Detection System (Applied Biosystems). The expression data was statistically analyzed using a one-tailed Students’ T-test with p<0.05 as significant.

## Results

### Discovery of Klf4 as a regulator of Pax6 with the enhancer element locator software

Enhancer Element Locator (EEL) was used to identify novel candidates for upstream regulators of Pax6. EEL is a program that searches over-represented transcription factor binding sites in the regulatory regions of a target gene [20]. It has been successfully used to identify transcription factor binding sites for c-Myc and N-Myc developmental signaling pathways [7]. In this study, with Pax6 set as the target gene, the EEL analysis reveals that Klf4 is in the top 5 most significant transcription factors. Klf4’s binding sites were in the top list of several other granule cell expressed genes such as Nfia, Cacna1a and Wnt7b. Thus, it was selected for experimental validation for its role in Pax6 regulation and cerebellum development.

### Characterization of Klf4 expression

**Fig 1:**
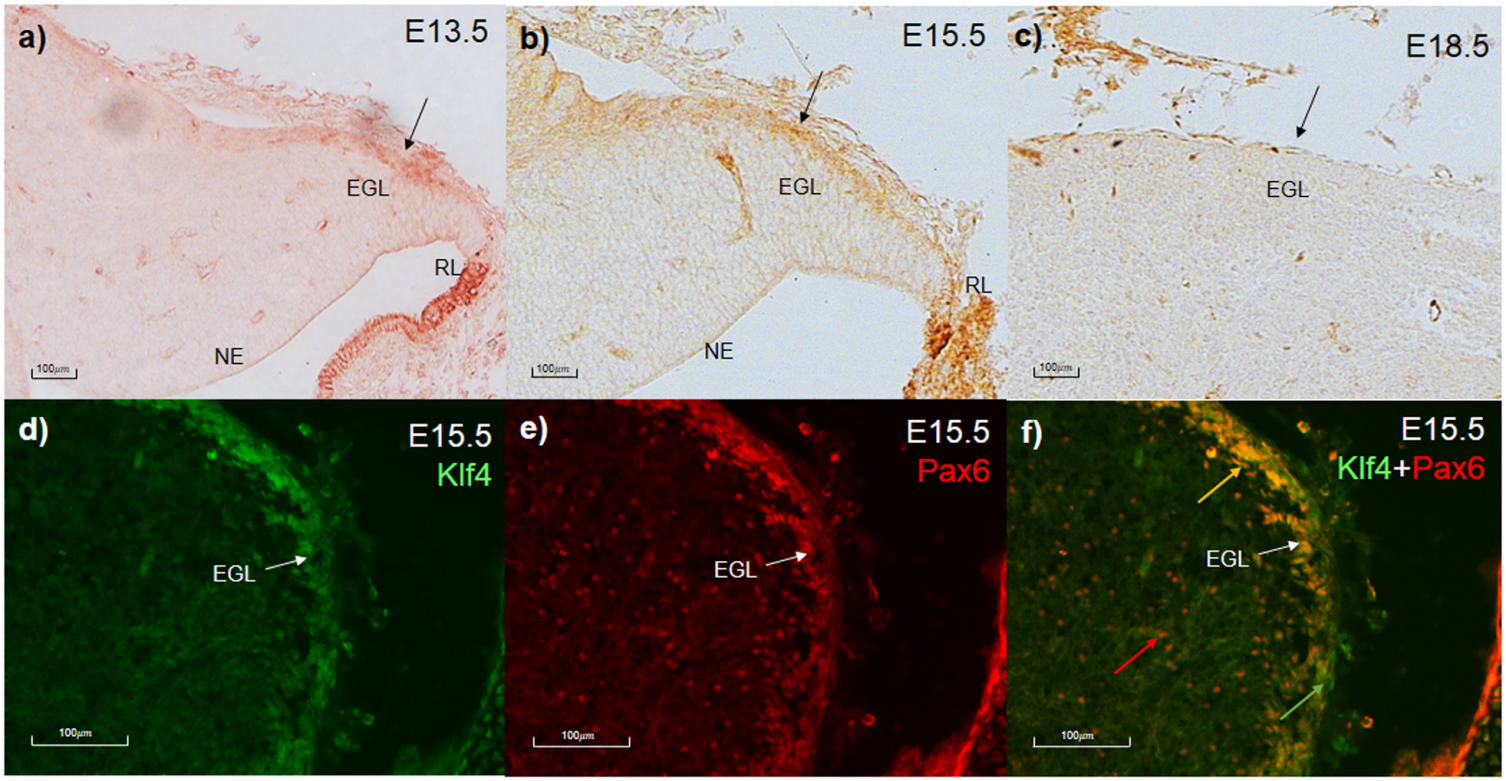
Klf4 expression in the cerebellum and its co-expression with Pax6. **a-c) Klf4 expression at: a) E13.5, b)E15.5, and c)E18.5**. Immunohistochemistry of Klf4 in the developing cerebellum. Klf4 is expressed in the EGL of the cerebellum at E13.5 and E15.5 but virtually no expression is seen at E18.5 (black arrows). **d-f) Co-expression of Klf4 and Pax6 in the EGL at E15.5**. Immunofluorescence staining of Klf4 (green, d), Pax6 (red, e) and merged picture (f) in the developing cerebellum. Klf4 and Pax6 are co-expressed in the EGL (white arrows) of the cerebellum at E15.5. In (f), green arrow indicates EGL cells that express only Klf4, red arrow indicates cells in the cerebellar core that express only Pax6, and yellow arrow indicates EGL cells that co-express Klf4 and Pax6. EGL- external granular layer, NE—neuroepithelium, RL- Rhombic lip.

To investigate Klf4 expression in the developing cerebellum, cerebellar tissues were collected from E13.5, E15.5 and E18.5 wild-type animals. RT-PCR and immunohistochemistry were performed on E13.5 and E15.5 cerebellar tissue to examine Klf4’s expression at RNA and protein levels, respectively. Klf4 is expressed in the wild-type cerebellum in E13.5, E15.5 and P0 (S1 Fig). Immunohistochemistry with anti-Klf4 was performed on E13.5 and E15.5 embryos. The staining in the cerebellum was much weaker than higher expressing regions such as the skin and the posterior rhombic lip (Fig 1b). Within the cerebellum, the main expression is in the cells of the EGL (Fig 1a). By E18.5, there was no detectible immunopositive staining for Klf4 (Fig 1c).

The expression of Klf4 in early developing EGL is co-incident with Pax6 expression[5]. To explore this possibility, we co-stained tissue with antibodies to Pax6 and Klf4. Almost all EGL cells were positive for Pax6 and Klf4 (Fig 1d and 1e). However, other cell populations that were Pax6-positive in deeper regions of the cerebellum were not Klf4-positive (Fig 1f).

### Pax6 in the developing cerebellum following the elimination of Klf4 expression

In addition to the identification of Klf4 binding sites upstream of Pax6, the immunocytochemical data suggest, an interaction between Klf4 and Pax6. To test this hypothesis more directly, we used a KO of the KLF4 gene to more mechanistically study a possible interplay between genes. The Klf4 knockout is a perinatal lethal and pups die after 4–6 hours after birth as previously reported [15]. Thus, we were limited in the examination of the developing cerebellum to prenatal and very early postnatal times.

**Fig 2:**
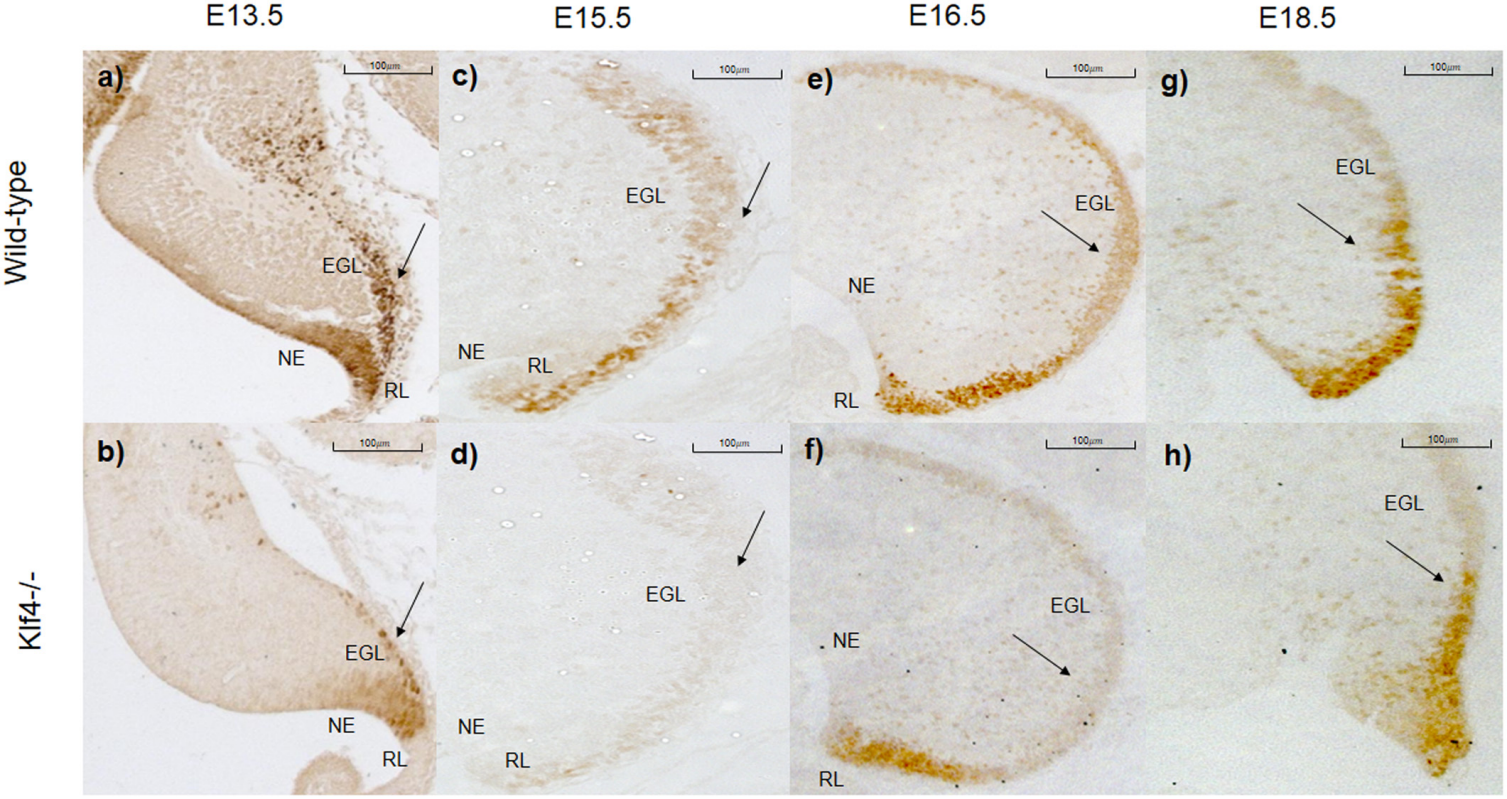
Pax6 is down-regulated in Klf4-null cerebellum at E13.5 and E15.5. Immunohistochemical demonstration of Pax6 expression during development in Klf4-wildtype (a,c,e,g) and -null (b,d,f,h) cerebellum. Pax6 immunocytochemistry is similar in the developing EGL of the wildtype cerebellum from E13.5 to E18.5. Pax6’s expression is greatly reduced at E13.5 and E15.5 in the Klf4-null cerebellum but rebounds at E16.5 and E18.5.

**Fig 3:**
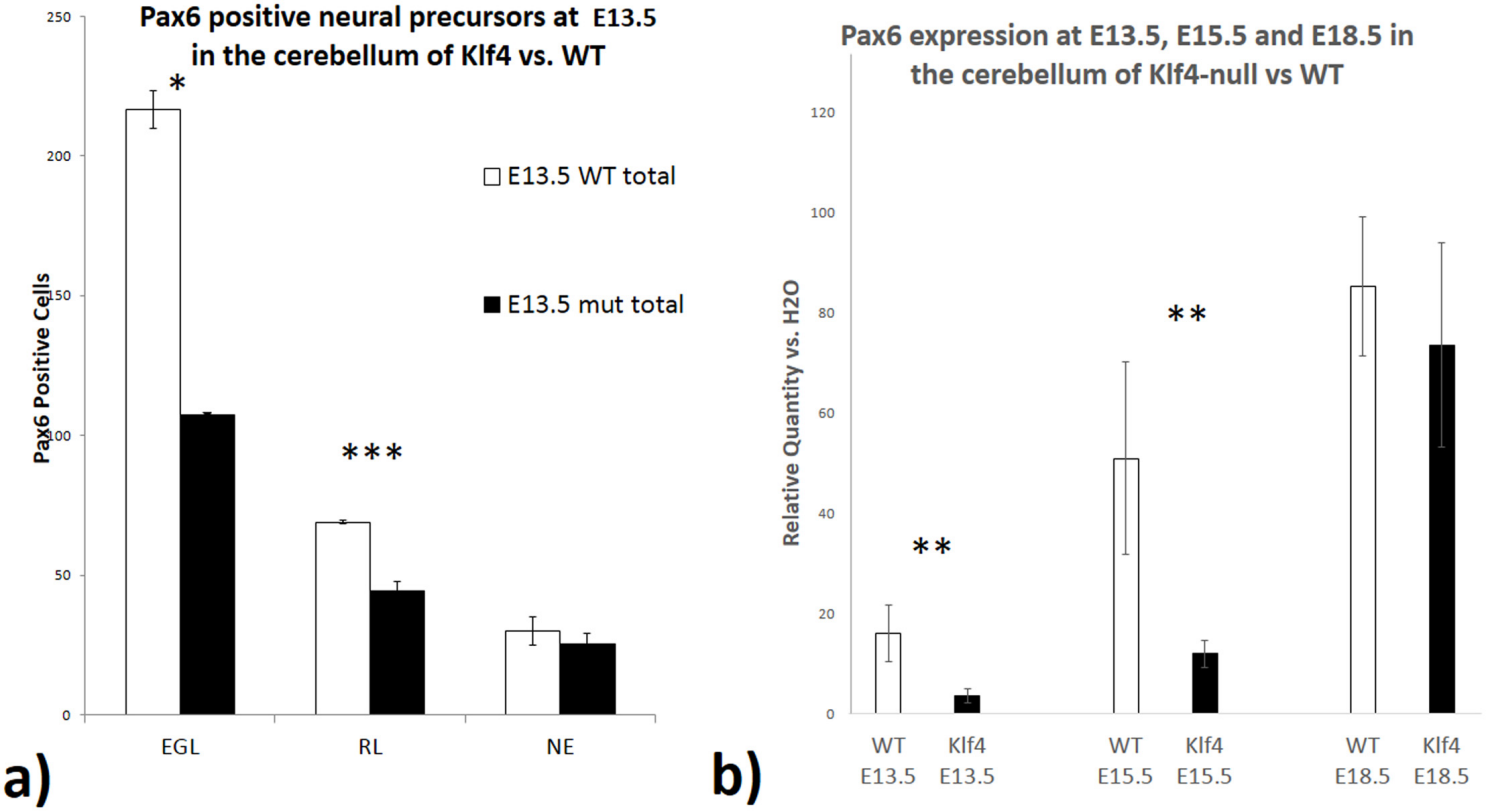
Quantification of Pax6 cell number and expression down-regulation in Klf4-null cerebellum. **3a)** E13.5 Pax6 positive granule cell count in Klf4-/- compared with wild-type in the EGL (p<0.05), RL (p<0.001), and NE. One-tail students’ T-test was used and results were represented with p<0.05(*), p<0.01 (**) and p<0.001 (***). **3b)** Real-time PCR showing the expression of Pax6 in the wild-type and Klf4-null at E13.5, E15.5 and E18.5 in the whole cerebellum. The expression of Pax6 is ~23% of the wild-type expression level in the Klf4-null in the E13.5 (p<0.01) and 15.5 (p<0.01). One-tail students’ T-test was used and results were represented with p<0.05(*), p<0.01 (**) and p<0.001 (***). Y-axis: Relative Quantity vs H2O –target gene expression of the sample compared against with a negative control where H2O were used as template. X-axis: EGL- external granular layer, NE—neuroepithelium, RL- Rhombic lip, WT- wild-type, Mut—Klf4-null.

We observe a marked reduction in Pax6 immunocytochemistry in Klf4-/- EGL cells at E13.5 (Fig 2a and 2b); and an almost complete elimination of staining in the E15.5 Klf4-null when compared to the wild type cerebellum (Fig 2c and 2d). These observations suggest that Klf4 positively regulates Pax6 expression. To examine if the reduction of Pax6 expression is due to a smaller number of cells expressing similar levels of Pax6 or the same number of cells expressing Pax6 at a lower level; we quantified the number of Pax6+ cells. Indeed, fewer Pax6-positive cells are found in the Klf4-/- cerebellum at E13.5 (Fig 3a, p<0.05). A similar reduction of Pax6+ cells is found in the rhombic lip (Fig 3a, p<0.001) but not the proliferative neuroepithelium above the 4^th^ ventricle. The quantitative assessment of Pax6 expression showed the similar reduction as seen in the sectioned and stained material at E13.5 (Fig 3b, p<0.01 and 15.5 (Fig 3b, p<0.01). Interestingly, at E16.5, there is a return of Pax6-immunopositivity in the EGL and RL (Fig 2e and 2f). The return of Pax6-positive staining appears almost complete by E18.5 (Fig 2g and 2h). The return of Pax6 expression at E18.5 is confirmed with real-time PCR (Fig 3b). Our observations indicate that Klf4 normally, positively regulates Pax6 during early granule cell development, prior to E16.5.

### Klf4’s roles in the developing cerebellum

To investigate what Klf4 might be doing in the developing cerebellum, we studied important developmental events, such as cell differentiation, cell death and cell proliferation, in the Klf4-/- cerebella using cell-specific markers: Gfap and Glast for glial cells, Calbindin for Purkinje cells, Pax2 for interneurons and Pax6 for granule cells. We did not observe any differences in the differentiation of Purkinje cells or cerebellar interneurons in the Klf4-null cerebellum. We also did not observe any differences in glial cell development in the Klf4-/- cerebellum (data not shown).

**Fig 4:**
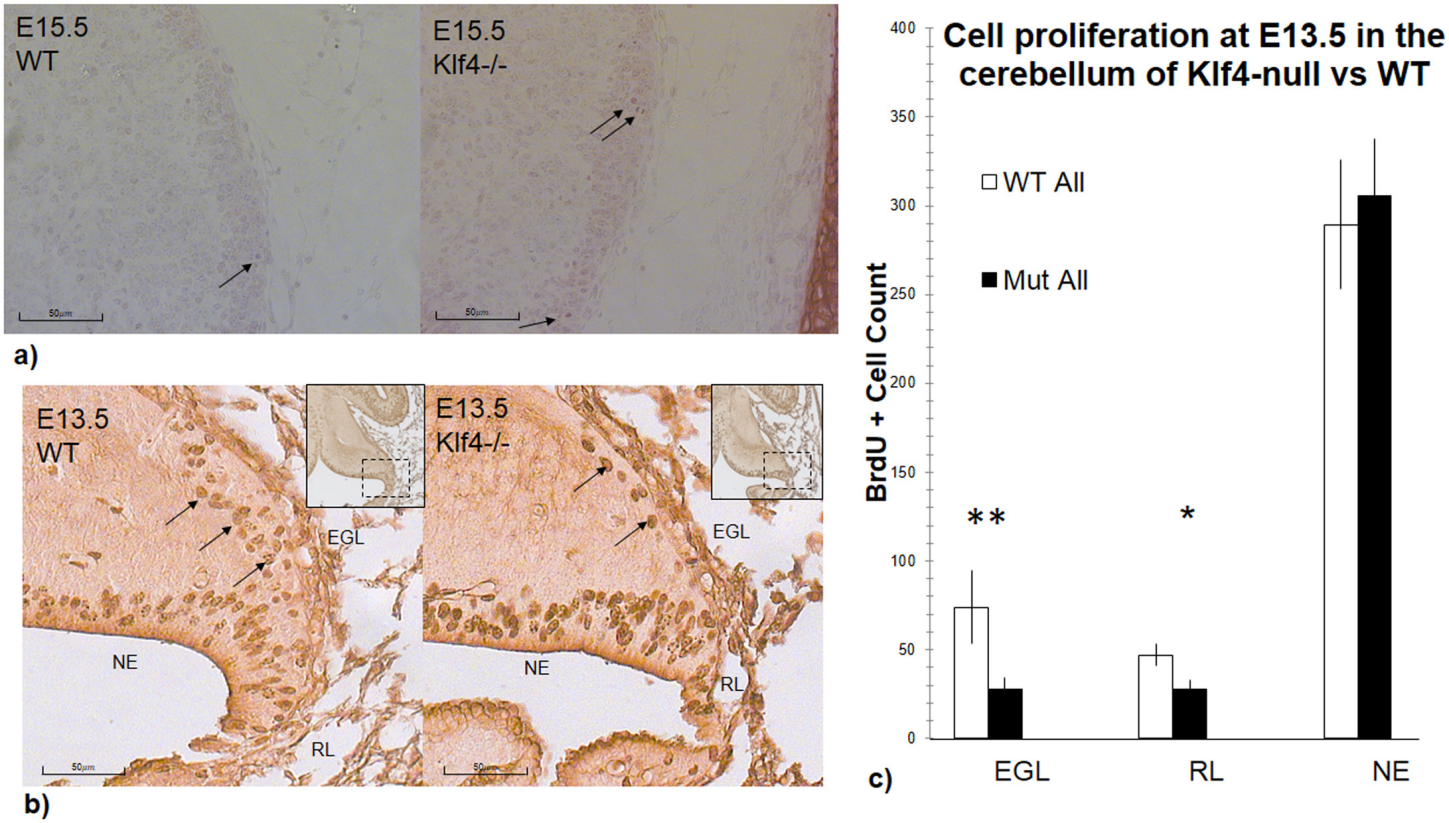
Effects of Klf4-knockout on cell death and/or cell proliferation in the developing cerebellum. **A) Cresyl-violet staining of wild-type and Klf4-null cerebellum**. The appearance of heterochromatic cells is a hallmark of the Klf4-null EGL compared to the wildtype at E15.5 (black arrows). The proliferating cells were identified as having condensed heterochromatin in one of the phases of mitosis. **b) and c) BrdU-staining demonstrates a reduced proliferation of EGL and RL cells in the Klf4-null at E13.5**. b) Immunolabeling of BrdU in the cerebellum and c) counting of BrdU+ cells at E13.5. Proliferative cells incorporate BrdU into newly synthesized DNA and become BrdU+. There is a decreased number of BrdU+ cells in the EGL (p<0.01) and RL (p<0.05) of the Klf4-null cerebellum. BrdU+ cells were identified as a dark brown staining after histochemical reaction with DAB. Number of BrdU+ cells were compared with one-tail students’ T-test and results were represented with p<0.05(*), p<0.01 (**) and p<0.001 (***). X-axis: EGL—external granular layer, RL-Rhombic lip, NE- neuroepithelium.

When we examined the wild-type and Klf4-null in Nissl stained material for gross cerebellar morphology, the size and general structure of the E13.5 and 15.5 mutant cerebellum are comparable to the wild-type. However, we observed more heterochromatic GCs in the Klf4-/- compared to its wild-type litter-mates suggesting a role of Klf4 in cell death and/or cell proliferation (Fig 4a). TUNEL and anti-Casp-3 immunostaining were performed to assess cell apoptosis in the Klf4-/- cerebellum. Few cells were undergoing apoptosis in either the wild-type or Klf4-/- cerebellum during early development; and no differences in apoptosis with TUNEL and Casp3 immunostaining were seen (data not shown).

**Fig 5:**
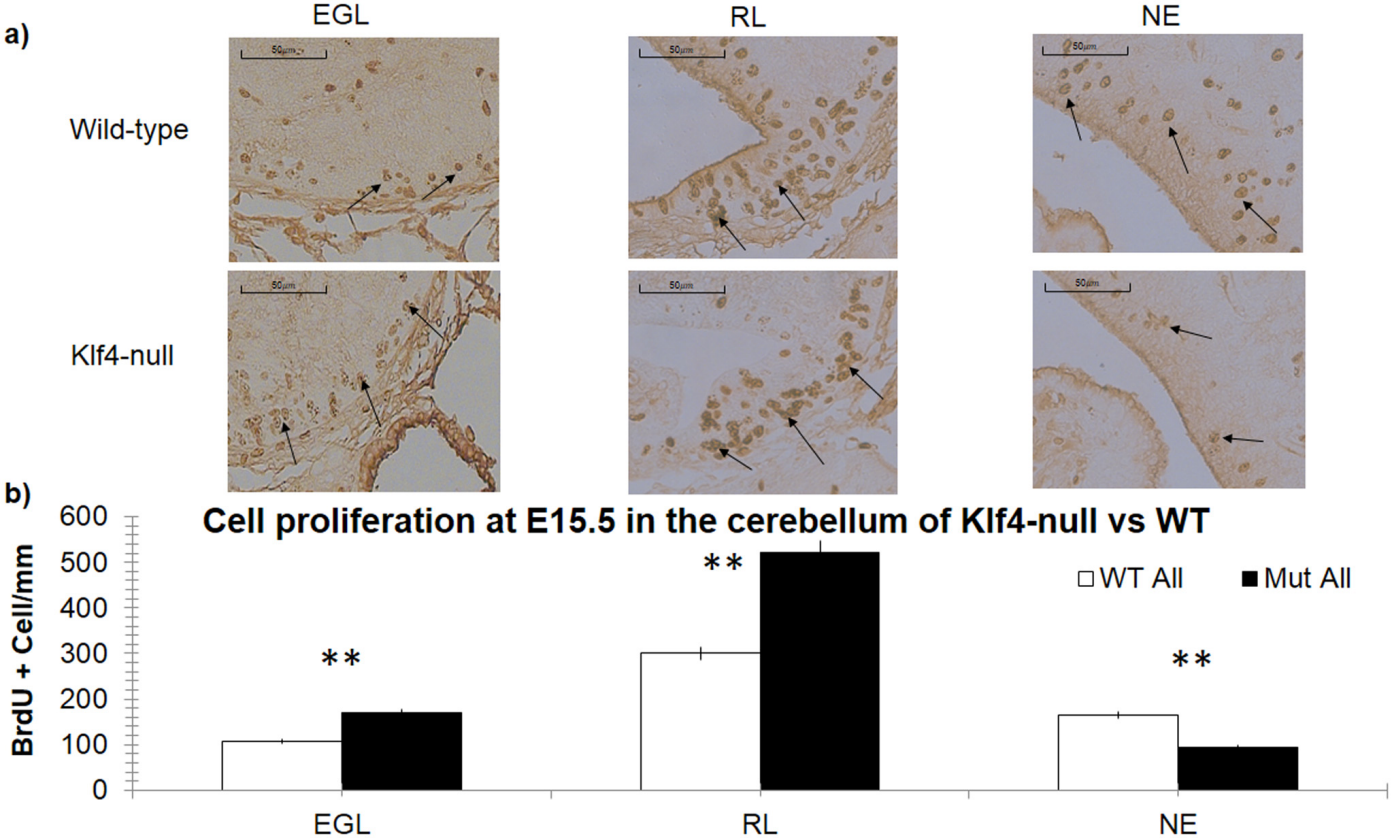
Klf4 has dual effects on the proliferation of epithelial cells in the cerebellum at E15.5. a) Immunolabeling of BrdU in the cerebellum and b) counting of BrdU+ cells at E15.5. There is an increased number of proliferating cells in the EGL (p<0.01) and RL (p<0.01), but a decreased number of proliferating cells in the NE (p<0.01) in the Klf4-null cerebellum, indicated by a one-tailed Students’ T-test p<0.05(*), p<0.01 (**) and p<0.001 (***). X-axis: EGL—external granular layer, RL-Rhombic lip, NE- neuroepithelium.

To look at cell proliferation, we used a short term (1 hour) BrdU exposure to assay cell proliferation in Klf4-null embryos. In the E13.5 Klf4-/- null cerebellum, we found a lower number of proliferating cells in the EGL (p<0.01) and RL (p<0.05) compared to wild-type litter-mates (Fig 4b and 4c). Furthermore, the EGL appears to be thinner and less extended in the Klf4-null (Fig 4b; see also Fig 2a and 2b). The reduced granule cell proliferation at E13 in the Klf4 -/- suggests a positive regulatory role of Klf4 on early granule cell proliferation. However, this proliferative effect of Klf4 in the EGL is reversed at E15.5 when more proliferating cells are found in the Klf4-null EGL (Fig 5, p<0.01) and RL (Fig 5, p<0.01). In addition, we also observed a decreased number of proliferating cells in the neuroepithelium (NE) where cerebellar interneurons are born at E15.5 (Fig 5b, p<0.01). This opposite effect on cell proliferation in the Klf4-null hints at a differential (either direct or indirect) regulation of Klf4 in the two cerebellar neurogenic regions.

### Investigation on functional redundancy in the Klf family

**Fig 6:**
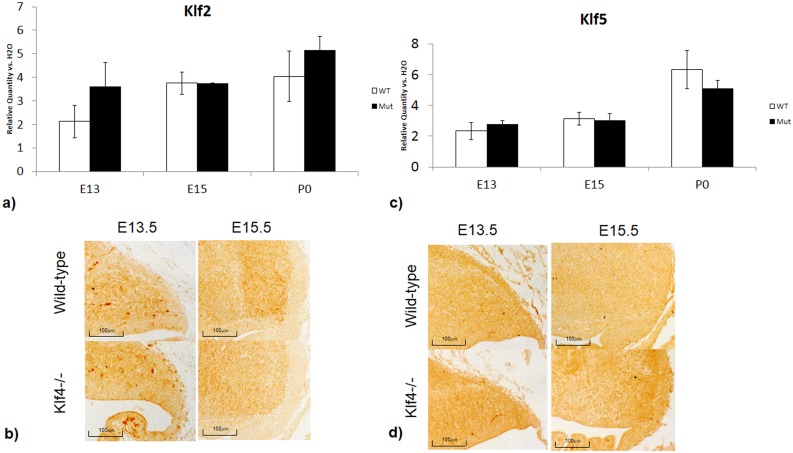
The expression levels of genes involved in complementary cell proliferative pathways in the Klf4-null with real-time PCR. a) RT-PCR and **b)** Immunohistochemistry showing Klf2 expression, a Kruppel-like factor belonging to the same gene family as Klf4, do not show expression changes in the Klf4-null. c) RT-PCR and **d)** Immunohistochemistry showing Klf5 a Kruppel-like factor belonging to the same gene family as Klf4, do not show expression changes in the Klf4-null. One-tail students’ T-test was used for analysis and results were represented with p<0.05(*), p<0.01 (**) and p<0.001 (***). Y-axis: Relative Quantity vs H2O –target gene expression of the sample compared against with a negative control where H2O were used as template. X-axis: WT- wild-type, Mut—Klf4-null.

**Fig 7:**
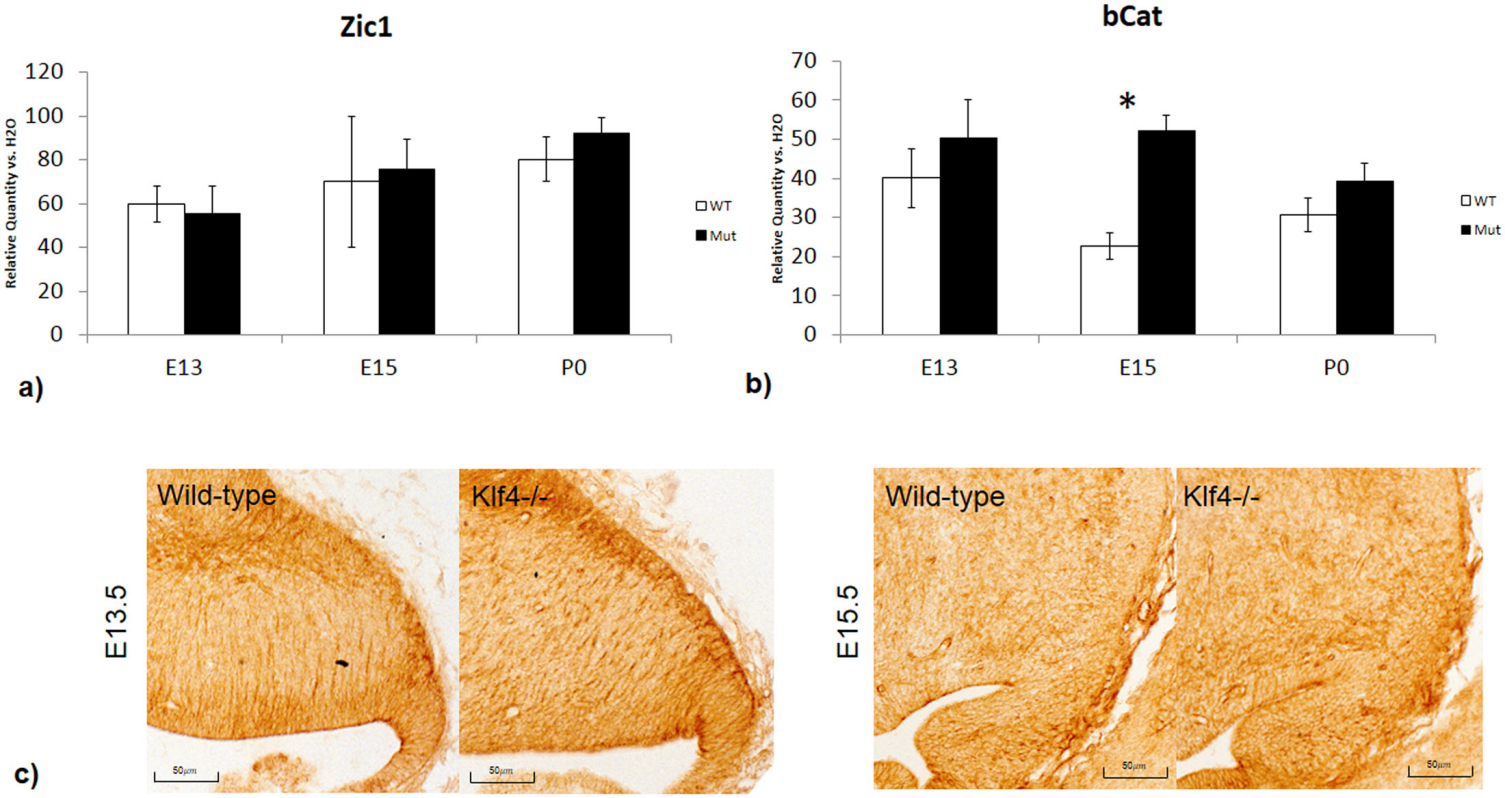
The expression levels of genes involved in alternative cell proliferative pathways in the Klf4-null with real-time PCR. **a)** Zic1, an early granule cell proliferation gene at E13.5, does not show expression changes in the Klf4-null. **b)** β-catenin, a member of Wnt pathway, shows an activated expression in the Klf4-null (p<0.05) at E15.5. E13.5 and E15.5 immunohistochemistry against β-catenin, there is no difference observed at E15.5 as the stain is generally weaker than E13.5. One-tail students’ T-test was used for analysis and results were represented with p<0.05(*), p<0.01 (**) and p<0.001 (***). Y-axis: Relative Quantity vs H2O –target gene expression of the sample compared against with a negative control where H2O were used as template. X-axis: WT- wild-type, Mut—Klf4-null.

While the findings on Pax6 expression and granule cell proliferation were robust in the Klf4-null at E13.5, the effects of Klf4 knockout were diminished at E18.5. One explanation for this result could be that the dynamic expression of Klf4 in the cerebellum—it is expressed highest in the cerebellum at E13.5 and lowest at E18.5. Thus, the Klf4-null phenotypes may be due to expression level differences over developmental time. Another possibility for the observed temporal differences in the Klf4-null is that the proliferative roles of Klf4 in the cerebellum could be replaced by other genes and pathways by E18.5. Other members of the Kruppel-like factor family are candidates for complete or partial functional redundancy since they are structurally similar. Therefore, we investigated potential functional redundant or functional complementary genes to Klf4. Expression level of Klf2 and Klf5, two other Klf transcription factors that have overlapping functions with Klf4 in the iPS cells, were not altered in the Klf4-/- at E13.5, E15.5 and P0 (Fig 6a and 6c). Immunohistochemistry staining at E13.5 and E15.5 also showed similar expression pattern for Klf2 (Fig 6b) and Klf5 (Fig 6d). The summarized expression data of all 17 Klf family members are shown in Table 1. Previous studies have shown that granule cell proliferation is regulated by at least two other molecular pathways: Zic and Wnt[21]. To examine these alternative granule cell proliferation pathways, the expression of the transcription factor Zic1 and β-catenin were measured with RT-PCR. We find that the expression level of Zic1 was normal in the Klf4-/- at E13.5 and E15.5 (Fig 7a). Gene expression of β-catenin at E13.5, determined by RT-PCR, showed a suggestive increase in the Klf4-/- when compared with wild-type, however, this increase was not significant (Fig 7b). Further validation with Anti-β-catenin immunohistochemistry showed that β-catenin is enhanced at E13.5 at the rhombic lip and EGL in the mutant compared to the wildtype (Fig 7c). At E15.5, there is a significant increase in β-catenin expression in the Klf4-null cerebellum (Fig 7b, p<0.05) determined by RT-PCR; however, this increased expression is not obvious with immunohistochemical staining of β-catenin at E15.5. At this time, the immunostaining of β-catenin in both Klf4-/- and wild-type is much weaker compared with staining at E13.5 (Fig 7c).

**Table 1:** The expression of Klf family members in mouse cerebellum.

*Gene Name*	In situ Cerebellar Expression Pattern (Genepaint)	Microarray expression level (CbGRiTS, normalized and averaged)	RIKEN deep-CAGE expression level (tpm, averaged)
***Klf1***	Not expressed	7.388083	0.32837
***Klf2***	Not expressed	8.130833	15.45146
***Klf3***	N/A	11.62117	25.8526
***Klf4***	Granule cells	7.47	2.814696
***Klf5***	Granule cells	6.819083	2.02686
***Klf6***	Not expressed	7.89725	15.89473
***Klf7***	Widespread	13.753	97.85526
***Klf8***	N/A	7.331333	3.614032
***Klf9***	Not expressed	10.59317	12.99584
***Klf10***	Granule cells	6.6785	15.35252
***Klf11***	N/A	6.5545	9.870974
***Klf12***	N/A	6.567083	0.473217
***Klf13***	Not expressed	10.45975	43.46916
***Klf14***	Not expressed	6.936	0.05531
***Klf15***	Purkinje cells	8.219917	8.684977
***Klf16***	N/A	8.642083	13.5992
***Klf17***	N/A	6.642333	0.031649

## Discussion

Klf4 is an important gene in many physiological and pathological processes, such as stem cell maintenance, skin development, cellular specification in the brain, and axon outgrowth[15,16,22,23]. The activation of Klf4 expression is found in immortalized kidney cells [24], laryngeal squamous cell carcinoma [24], ductal carcinoma of the breast [25] and skin carcinoma [26]. The activation of cell cycle by Klf4 could involve the repression of p53 pathway, which is a critical check point for cell cycle [27,28].

Our current study shows that Klf4 regulates early granule cell proliferation and could positively regulate transcription factor Pax6. Similar to its positive role in promoting self-renewal of embryonic stem cells, Klf4 expression is important for granule cell proliferation at E13.5 in the cerebellum. This aligns with recent expression data using Cap-associated transcriptome sequencing that shows the highest expression of Klf4 in the cerebellum is found at embryonic day 13 [19].

Importantly, Klf4-null showed a decreased number of Pax6+ EGL cells as well as a decreased proliferation of these cells at E13.5 which likely resulted in the less extensive (in the caudal-to-rostral dimension) and thinner (in the dorsal to ventral dimension) EGL during early development. These data suggest that the expression of Klf4 is important to cerebellar development and generation of granule cells.

### Klf4 regulates Pax6 expression

Our bioinformatic analysis showed that Klf4 is a potential upstream regulator of Pax6. The regulation of Pax6 by Klf4 has been demonstrated at the expression and phenotypic levels in eye development [9,10]. The expression of Pax6 is lowered to about half of its normal level in the cornea when Klf4 is conditionally knocked-out [9]. In addition, the defective corneal phenotype of the Klf4 knock-out resembles that of the heterozygous Pax6 knock-out [10]. Direct binding of Klf4 at Pax6 regulatory region has been found with genome-wise ChIP-seq analysis using mouse embryonic stem cells[29]. Therefore, we were interested to see if the expression of Pax6 is disrupted in the Klf4 knockout. Indeed, Pax6 expression is dysregulated in the EGL of the developing cerebellum in the Klf4-null. In the Klf4-null, the expression of Pax6 is greatly reduced at E13.5 and E15.5, indicating a positive regulation of Klf4 on Pax6. This observation is consistent with the role of Klf4 on Pax6 during corneal development where the Klf4-null showed about 50% Pax6 expression of a wild-type control[10].

However, our phenotypic data indicate that the Klf4-null granule cell is distinct from the Pax6-null granule cell; e.g., the Pax6-null phenotype is associated with deficits in neurite extension and cell migration, and a thickening of the EGL [5,30]. None of these phenotypes are seen in the Klf4-null cerebellum. Two cerebellar phenotypes that we see in the Klf4-null, however, are not observed in the Pax6 mutant: reduced cell proliferation in the EGL [5,9,15] and the shorter, thinner EGL at E13.5. In summary, despite that expression of Pax6 is partially abolished in the Klf4-null, the Klf4-null phenotypes we observed were distinct from either Pax6-null or Pax6+/- (which are phenotypically normal). This leads us to suggest that the phenotypes we observed in the Klf4-null cerebellum are independent of Pax6.

Finally, to examine the interplay between Klf4 and Pax6, we examined expression of Klf4 in the Pax6-null cerebellum in our Cb-GRiTS database [18]. We did not see a difference in Klf4 expression in the Pax6-null at E13.5, E15.5, or E18.5. This suggests that Klf4 is upstream of Pax6 in terms of transcriptional activation and this regulation could be direct binding of Klf4 to the promoter region of Pax6 [29].

### Klf4 as a regulator of cell proliferation

A key question in this study is what Klf4 is doing in the developing EGL during early cerebellar development. Previous studies demonstrated that Klf4 may serve either a role as a transcription activator or repressor depending on the gene targets and other co-factors; thus, it could either promote or inhibit cell proliferation under different cellular contexts. With BrdU labeling, we were able to show that in the Klf4-null, cell proliferation was up-regulated within the EGL and rhombic lip at E15.5. This suggests that Klf4 regulates a different set of gene targets at different developmental stages in the granule cells. Previous work has indicated the Wnt pathway as important to proliferation during granule cell development after E15, and Klf4 can inhibit Wnt signaling by directly interacting with β-catenin and TCF-4 [31]. Indeed, we observed an increased expression of β-catenin in the Klf4-/- at E15.5 indicating an inhibitory role of Klf4 on granule cell proliferation through Wnt signaling at E15.5. The activation of the Wnt pathway by Klf4 at E15.5 could be indirect and serve as an internal “rescue” in the Klf4 null cerebellum to remedy the early loss of granule cell precursors at E13.5. The “rescue” is seen by a normal level of total and proliferating granule cells, in the E18.5 Klf4-null cerebellum. However, at E13.5 when we see an EGL proliferation deficit in the Klf4-null it is not likely to involve Wnt signaling as signaling is not activated until E15.5 [21]. A myriad of other molecular partners for Klf4 function have been identified through whole genome chromatin immune-precipitation work; identifying more than 1,800 loci in human embryonic stem cells that are directly bound by Klf4 –many of these genes, such as Oct4 and Nanog are important transcription factors in cell proliferation [29].

We also see a cell proliferation phenotype in the NE of the Klf4-null cerebellum. Interestingly, this phenotype is in the opposite direction of the EGL cell phenotype; that is the cells of the neuroepithelium (NE) demonstrated decreased cell proliferation in the E15.5 cerebellum. However, from our data, Klf4 is not measurably expressed in the NE. In any case, it is of interest that these two proliferative regions give rise to two distinct cell types based upon neurotransmitter phenotype [4], and these two cell classes have mutually exclusive neuronal markers throughout cerebellar development[32]. Thus, while the glutamatergic granule cells are generated from the rhombic lip, the GABAergic neurons of the cerebellum are generated from the NE, located at roof of the fourth ventricle, specified by the basic helix-loop-helix (bHLH) transcription factor, Ptf1a[33]. The Klf4-null showed a reduction in the number of proliferating cells in the NE at E15.5. At this time point, several types of interneurons, such as the Golgi cells and basket cells, are generated at the NE; these cells populate the molecular layer and provide an inhibitory input to PCs in the mature cerebellum[34]. The pro-proliferation effect in the NE and the anti-proliferation effect in the EGL of Klf4 observed at E15.5 could be the results of Klf4 acting in different neural progenitors; or that the proliferation of one germinal zone is secondary to the other. This cross-germinal-region effect on cell proliferation further suggests the importance and complexity of Klf4 during early cerebellar development.

In addition to cell proliferation, we also examined other important developmental processes in the Klf4-null. Klf4 has been previously identified as a key gene for cell differentiation, such as in the granular cells in the skin [15], goblet cells in the small intestine [14] and neurons in the cerebral cortex [35]. Klf4 has also been identified to affect apoptosis in various cancers, mostly due to its interaction with p53 [8]; however, the role of Klf4 in apoptosis was not reported in the developing skin, eye and intestine of the KLf4 knockout mouse [9,14,15]. Furthermore, Klf4 also regulates gliogenesis in the cerebral cortex by directly interacting with CBP/p300 [36]. We did not observe any changes in apoptosis and gliogenesis during cerebellar development in Klf4-null.

### Temporal specificity of Klf-null phenotype

The phenotype of the Klf4-null cerebellum during early development were not observed at E18.5 or P0. We were intrigued to understand what might be responsible for the normal phenotype at later developmental stages in the knockout. Three possibilities emerged as candidates for altered phenotypic expression over time. First, this may be due to the temporal expression of Klf4 which is heightened at E13.5 but falls off later in embryonic development. Second, as discussed above, alternative molecular pathways could be responsible at different points in time. Third, other members of the Klf4 family could be substituting for the absence of Klf4 at later times but not earlier. Expression databases indicate that multiple members of the Klf family of transcription factors are frequently coexpressed and may have redundant functions. Functional redundancy among different Klf family members has been previously observed—e.g., Klf2 and Klf5 shared function with Klf4 in the stem cells so that cell cycle arrest only occurs when all three factors are knocked out[37]. We examined expression of Klf2 and Klf5 in the Klf4-/-, and no significant changes in expression were found with these family members. However, there are 14 other Klf factors that we did not investigate and could share similar DNA binding sequences with Klf4. It will be interesting in future work to tease out the unique or overlapping functions of these Klfs in the granule cells during cerebellar development. Currently, we favour the second possibility as the most parsimonious explanation of the temporal specificity of the Klf4-null phenotype in cerebellar granule cells.

### Comparison of the Klf4-null with the Pax6-null cerebellum

A key question in our analysis is whether Klf4 has a unique role, other than its interplay with Pax6 in cerebellar development. To address this question a comparison of cerebellar phenotypes between Klf4-null and Pax6-null is informative. While the structures of the Klf4-null and Pax6-null, cerebellum are apparently normal at E13.5, the Klf4-null is differentiated from the Pax6-null in that the EGL is thinner and less extended. In addition, later in development, the Klf4-/- EGL shows an altered cell proliferation but normal migration and foliation, whereas Pax6-/- was normal in this regard but showed aberrant differentiation, migration and foliation [30]. These differential phenotypes may be due to differences in Pax6 expression in the Klf4-null (30% of wild-type) compared to the Pax6-null (virtually 0%). In fact, the cerebellum of the Pax6+/- (with 50% functional Pax6 molecules) appears normal [30]. This could suggest a dosage effects of Pax6 in cerebellar development. On the other hand, the phenotypic differences could reflect Klf4 and Pax6’s complex functions in transcriptional activation and/or suppression in the developing cerebellum.

## Conclusions

Klf4 regulates early granule cell proliferation in a time-specific manner: at E13.5, Klf4 promotes granule cell proliferation through a pathway that is apparently independent of Zic1; whereas at E15.5, Klf4 showed an inhibitory role on granule cell proliferation, possibly through the suppression of the canonical Wnt pathway. Klf4 also positively regulates Pax6, this regulation might be direct as a Klf4 binding site has been found upstream of Pax6 in previous chip-seq studies. The next steps are to more fully elucidate the regulatory network involving Klf4 in developing cerebellar granule cells.

## Supporting Information

S1 Fig Klf4 expression in wild-type and Klf4-null cerebellum. Klf4 is expressed in wild-type cerebellum at E13.5, E15.5 and P0. Its expression is greatly abolished in the Klf4-null. Y-axis: Relative Quantity vs H2O –target gene expression of the sample compared against with a negative control where H2O were used as template. X-axis: WT- wild-type, Mut—Klf4-null.
(TIF)
