# Supplementary figures and images for: Kruppel-Like Factor 4 Regulates Granule Cell Pax6 Expression and Cell Proliferation in Early Cerebellar Development
Source: PLoS One. 2015 Jul 30;10(7):e0134390. doi: 10.1371/journal.pone.0134390 (PMC4520560; doi:10.1371/journal.pone.0134390)

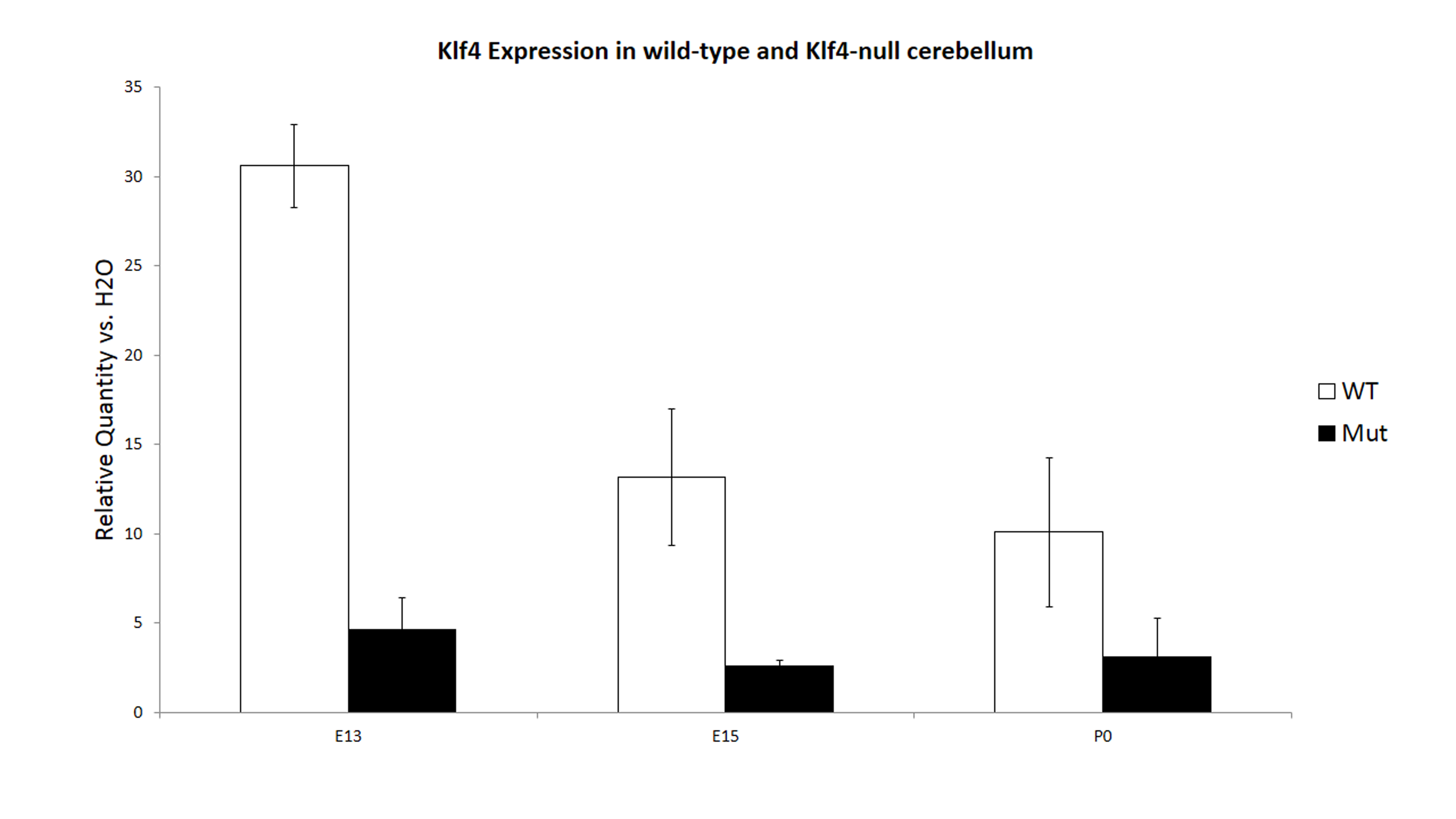

Supplement: S1 Fig — Klf4 is expressed in wild-type cerebellum at E13.5, E15.5 and P0. Its expression is greatly abolished in the Klf4-null. Y-axis: Relative Quantity vs H2O –target gene expression of the sample compared against with a negative control where H2O were used as template. X-axis: WT- wild-type, Mut—Klf4-null. (TIF) [file pone.0134390.s001.tif]
